# Supplementary material for: Topologically cloaked magnetic colloidal transport
Source: Nat Commun. 2025 Feb 20;16:1828. doi: 10.1038/s41467-025-57004-4 (PMC11842733; doi:10.1038/s41467-025-57004-4)
Supplement: Supplementary file 1 — Description of Additional Supplementary Files [file 41467_2025_57004_MOESM1_ESM.pdf]

Description of Additional Supplementary Files
Topologically cloaked magnetic colloidal transport

Anna M. E. B. Rossi,[1] Thomas Märker,[1] Nex C. X. Stuhlmüller,[1] Piotr Kuświk,[2] Feliks Stobiecki,[2] Maciej Urbaniak,[2] Sapida Akhundzada,[3] Arne J. Vereijken,[3] Arno Ehresmann,[3] Daniel de las Heras,[1] and Thomas M. Fischer[1,*]

[1]*Institute of Physics, Universität Bayreuth, 95440 Bayreuth, Germany.*
[2]*Institute of Molecular Physics, Polish Academy of Sciences, 60-179 Poznań, Poland.*
[3]*Institute of Physics and Center for Interdisciplinary Nanostructure Science and Technology (CINSaT), University of Kassel, D-34132 Kassel, Germany*
(Dated: January 31, 2025)

This PDF file includes:
Description of Supplementary Video 1 to 6
Description of Supplementary Data 1

MOVIE FILES

File Name: Supplementary Video 1
Description: Supplementary video 1 shows the dynamics of the colloidal ensemble traveling around the diamond shaped cloak.

File Name: Supplementary Video 2
Description: Supplementary video 2 shows the opening of the boat-shaped cloak and the associated broadening of the fence points in control space as we increase the cloak size R.

File Name: Supplementary Video 3
Description: Supplementary video 3 shows the dynamics of the colloidal ensemble traveling around the circular shaped cloaks of various sizes.

File Name: Supplementary Video 4
Description: Supplementary video 4 shows the dynamics of the colloidal ensemble traveling around the boat shaped cloaks of various sizes.

File Name: Supplementary Video 5
Description: Supplementary video 5 shows the dynamics of the colloidal ensemble traveling around the rounded square shaped cloaks of various sizes.

File Name: Supplementary Video 6
Description: Supplementary video 6 shows the dynamics of the conformation of eight colloidal particles traveling around a boat-shaped cloak. The conformation of the particles is restored after passing the cloak.

File Name: Supplementary Data 1
Description: All trajectories of particles are extracted from Supplementary Videos 3-5. We append the tracked particle files and a python code in a supplementary data set called Supplementary Data 1 that converts this data into figures 3 and 4.
